# Primary Hyperparathyroidism in Celiac Disease: Diagnostic Challenges and Therapeutic Strategies

**DOI:** 10.3390/nu18183107

**Published:** 2026-09-21

**Authors:** Quyen V. Luong, Kadiyatu Fofana, Abdul W. Paracha, Ayesha Siddiqui, Kofi Clarke

**Affiliations:** 1 Department of Medicine, Division of Endocrinology, Diabetes and Metabolism, Penn State Health Milton S. Hershey Medical Center, Penn State College of Medicine, Hershey, PA 17033, USA; qluong@pennstatehealth.psu.edu (Q.V.L.); kfofana@pennstatehealth.psu.edu (K.F.); asiddiqui1@pennstatehealth.psu.edu (A.S.); 2 Michael E. DeBykey Department of Surgery, Baylor College of Medicine, Houston, TX 77030, USA; abdulwasay.paracha@bcm.edu; 3 Department of Medicine, Division of Gastroenterology and Hepatology, Penn State Health Milton S. Hershey Medical Center, Penn State College of Medicine, Hershey, PA 17033, USA

**Keywords:** celiac disease, primary hyperparathyroidism, secondary hyperparathyroidism, parathyroid adenoma, gluten-free diet, normocalcemic hyperparathyroidism, tertiary hyperparathyroidism, quaternary hyperparathyroidism, vitamin D

## Abstract

Celiac disease (CD) is a primary immune-mediated enteropathy triggered by gluten ingestion in genetically predisposed individuals. Gluten ingestion results in villous atrophy of the small intestine and can lead to impaired absorption of multiple vitamins and nutrients, including vitamin D and calcium. This may result in vitamin D deficiency, a physiologic stimulus for parathyroid hormone (PTH) secretion, subsequently causing secondary hyperparathyroidism. Malabsorption and hyperparathyroidism in CD can contribute to metabolic bone disease (MBD), including osteopenia or osteoporosis. Correction of CD with a gluten-free diet (GFD) does not always reverse MBD. Although malabsorption in CD is a well-recognized cause of secondary hyperparathyroidism, some case reports have also suggested a potential association between CD and primary hyperparathyroidism (PHPT). In this literature review, we present evidence from observational studies, case series, and case reports that suggest a potential association or coexistence of CD and PHPT. Although the exact relationship between CD and PHPT remains unclear, limited evidence suggests that the two conditions may be related or may coexist independently of one another, with GFD treatment potentially unmasking underlying PHPT in patients with CD. One proposed mechanism is that vitamin D deficiency in CD reduces the vitamin D-mediated suppression of parathyroid cell proliferation, potentially contributing to the development of PHPT; however, current evidence remains insufficient to establish a causal relationship. PHPT and CD can contribute to MBD with increased fracture risk and adversely affect quality of life and overall health. Monitoring PTH, vitamin D, and calcium levels is important to identify persistent hyperparathyroidism and unmask underlying PHPT after correction of malabsorption. The literature review highlights a gap in our understanding of the pathogenesis linking these two conditions and underscores the need for future studies to elucidate their potential relationship. Because surgery is the only curative treatment modality for PHPT, patients with persistent hyperparathyroidism despite correction of malabsorption should be evaluated for possible concomitant PHPT, as they may require interdisciplinary management involving gastroenterologists, endocrinologists, and endocrine surgeons.

## 1. Introduction

Celiac disease (CD) is a primary immune-mediated enteropathy triggered by gluten ingestion in genetically predisposed individuals [1]. It is estimated that about 3 million people in the United States have CD; however, approximately 60 to 70% of Americans with CD are not diagnosed. The estimated global prevalence is about 1 in 100 people worldwide [2,3]. CD is associated with vitamin and micronutrient deficiencies as well as metabolic bone disease (MBD). It can also present with other autoimmune conditions such as type 1 diabetes mellitus, Hashimoto’s thyroiditis, Graves’ disease, Addison’s disease, rheumatoid arthritis, autoimmune hepatitis, etc.

In susceptible individuals, gluten exposure induces immune-mediated villous atrophy of the small intestine, causing impaired nutrient absorption, resulting in malnutrition and other complications. One important aspect of CD care and follow-up, with ongoing debate and evolving literature, is the monitoring of bone health. Patients with CD are at high risk of developing osteoporosis, a condition where bone mineral density (BMD) loss is severe and predisposes patients to fractures, leading to poor quality of life and other comorbidities. Osteoporosis in this setting has been commonly attributed to secondary hyperparathyroidism caused by vitamin D malabsorption. However, over the past 2–3 decades, several cases of primary hyperparathyroidism (PHPT) in conjunction with CD have been reported, suggesting an association between these two seemingly unrelated conditions.

In this review, we explored reported cases describing the association between CD and PHPT. Given the limited data on the potential shared pathogenesis of these conditions, evidence regarding the underlying mechanisms linking them is yet to be explored. This review highlights an important gap in our understanding of the relationship between CD and PHPT, which warrants further investigation in future studies.

## 2. Literature Search

A literature search was conducted in the PubMed database to identify journal articles reporting on the coexistence of PHPT and CD. The search was limited to articles published in English between January 2001 and August 2025. The search strategy included the following keywords: “celiac disease,” “primary hyperparathyroidism,” “bone disease,” “gluten-free diet,” “parathyroid hormone (PTH),” and “osteoporosis.” Journal articles describing patients with CD who subsequently developed or were diagnosed with PHPT were identified. Our literature search was not restricted to a certain age group or population, and case reports with incomplete information were not excluded. Although this is likely not an exhaustive list, as the case reports were obtained from only one database, it represents a broad spectrum of patients affected by CD who may have coexisting PHPT and bone complications.

## 3. Celiac Disease and Primary Hyperparathyroidism

Although CD is well known to be associated with secondary hyperparathyroidism due to malabsorption and vitamin D deficiency, an association between CD and PHPT has also been reported over the past few decades. In the case series by Maida et al., the prevalence of PHPT has been reported to be 2.3% in patients with CD [4], which is higher than the estimated prevalence of PHPT in the general population (0.84–1.02%) [5]. All patients in this case series were reported to have a single-gland parathyroid adenoma, rather than hyperplasia that is usually seen in secondary hyperparathyroidism. Ludvigsson et al. studied a larger cohort of 17,121 patients with CD, using a large population-based sample, and found a 2-fold increased risk of PHPT in patients with CD, especially in the first 5 years of diagnosis (Hazard Ratio 1.91, 95% CI 1.44–2.52) [6]. In a recent retrospective study of adult patients undergoing evaluation for an initial parathyroidectomy for presumed sporadic PHPT, approximately 6% of the 11% of patients who were screened for CD due to the presence of gastrointestinal symptoms had positive CD serology (tissue transglutaminase immunoglobulins, endomysium antibodies, and deamidated gliadin peptide immunoglobulins) [7]. These findings further highlight an association between CD and PHPT; however, the true prevalence of CD in this population was difficult to determine given the retrospective study design and the limited proportion of patients who underwent CD screening.

Most case reports on PHPT and CD mention the presence of parathyroid adenoma, rather than simple parathyroid hyperplasia. Parathyroid adenoma is the most common presentation in PHPT (85%), followed by multiglandular involvement (15%), and parathyroid carcinoma (<1%) [8]. Elevated parathyroid hormone (PTH) levels are commonly observed in patients with CD as a result of secondary hyperparathyroidism, which may mask or delay the diagnosis of PHPT. Interestingly, in these case reports, most patients were diagnosed with PHPT after being started on a gluten-free diet (GFD) and in a state of controlled or treated CD. It has been proposed that vitamin D deficiency resulting from malabsorption in patients with CD may lower serum calcium levels into the normal range, as vitamin D plays a critical role in intestinal calcium absorption. Subsequent treatment with GFD improves calcium and vitamin D absorption, which may reveal the underlying hypercalcemia and lead to the diagnosis of PHPT. Thus, GFD in CD patients can unmask PHPT after repletion of vitamin D stores. Another proposed hypothesis is that small intestinal inflammation in CD due to increased titer of autoantibodies can trigger PHPT in susceptible individuals, and treatment of CD with GFD causes gradual healing of intestinal mucosa, leading to eventual decline in the risk of PHPT development [6].

**Figure 1:**
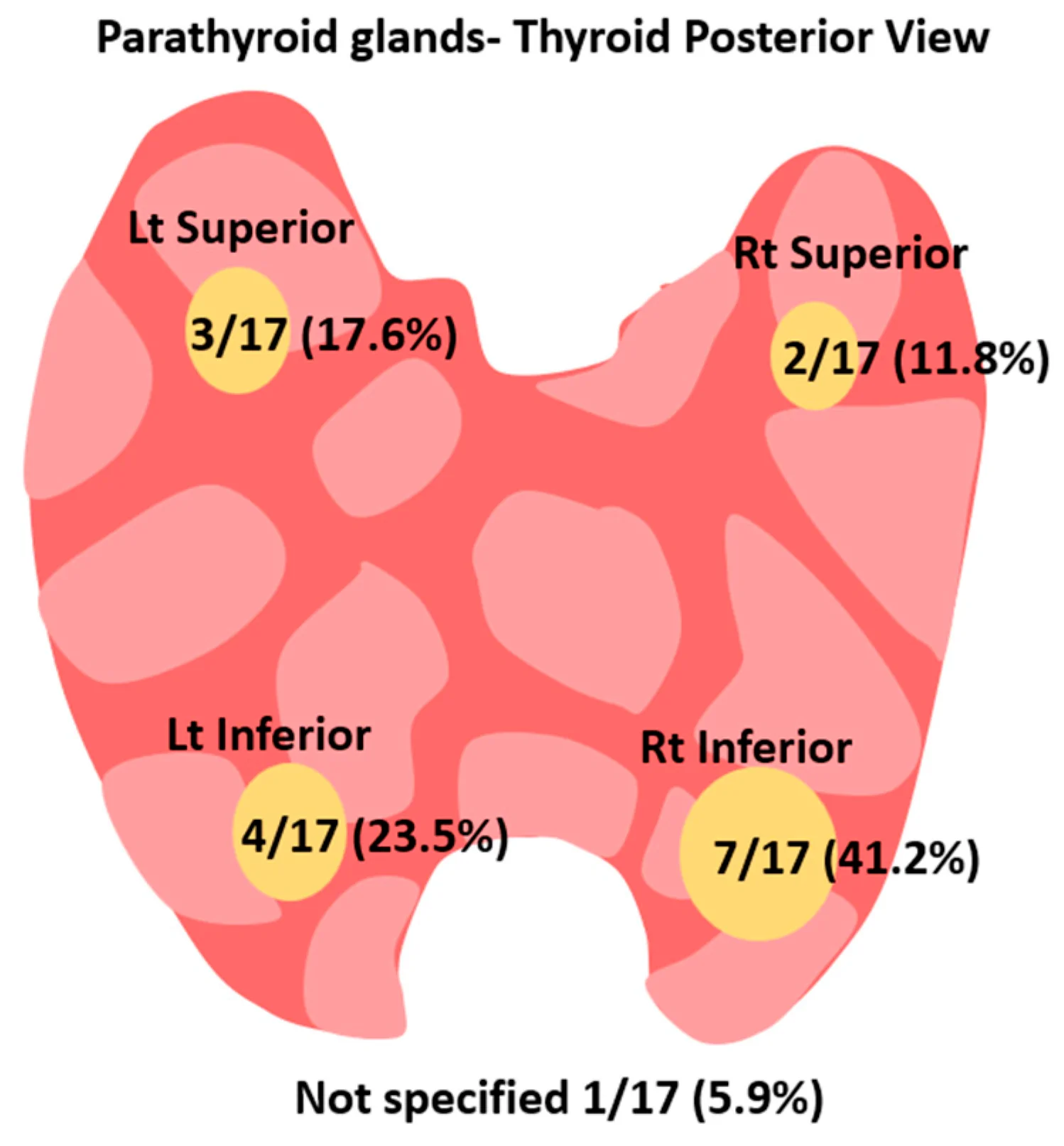
Distribution of parathyroid adenomas in reported patients with CD and PHPT. Lt: left; Rt: right. A total of seventeen patients were reported in the identified case reports. There was one patient for whom the adenoma location was not specified. The percentages were calculated including the patient with parathyroid adenoma location not specified. Observed parathyroid adenomas in these case reports—left superior: 3; right superior: 2; left inferior: 4; right inferior: 7.

In the reported cases, several patients presented with severe fatigue and fragility fractures, which led to evaluation of secondary causes of osteoporosis, including CD and PHPT. Sestamibi parathyroid scan and neck ultrasound in these cases demonstrated the presence of a single enlarged parathyroid gland, which suggests a parathyroid adenoma rather than secondary hyperparathyroidism. Analysis of the reported cases showed that the majority of patients had a parathyroid adenoma located in the right inferior gland (41.2%), followed by the left inferior (23.5%), left superior (17.6%), and right superior (11.8%) glands (Figure 1). A summary of the reported cases is presented in Table 1.

## 4. Mechanisms

Several hypotheses have been proposed to explain the association between CD and the development of parathyroid adenomas, including the loss of the suppressive effects of vitamin D on parathyroid cell proliferation, independent coexistence of the two conditions, and autoimmune mechanisms. Vitamin D, particularly its active metabolite 1,25-dihydroxyvitamin D, suppresses parathyroid cell proliferation and inhibits parathyroid hormone gene transcription, thereby reducing PTH synthesis and secretion [18]. CD leads to vitamin D malabsorption, thereby reducing the inhibitory effects of vitamin D on parathyroid proliferation, which may promote the expression of proto-oncogenes implicated in parathyroid tumorigenesis. Additionally, the calcium-sensing receptor (CaSR), a key regulator of PTH secretion in response to extracellular calcium, has been implicated in the pathogenesis of primary hyperparathyroidism. Reduced CaSR expression has been demonstrated in hyperplastic parathyroid glands and parathyroid adenomas [19,20]. Because CaSR downregulation is not caused by hypercalcemia alone [19], the reduced expression is likely attributable to other intrinsic factors. Indeed, decreased CaSR sensitivity or defective CaSR raises the calcium set point required to suppress PTH secretion, resulting in sustained PTH secretion characteristic of primary hyperparathyroidism [21].

A prolonged course of untreated or poorly controlled CD with associated vitamin D and calcium malabsorption can eventually lead to tertiary hyperparathyroidism, wherein parathyroid glands undergo hyperplasia with subsequent autonomous PTH secretion. Although the term “quaternary hyperparathyroidism” has been proposed, it is not a widely recognized concept or established clinical entity. Brain et al. described quaternary hyperparathyroidism as the progression from autonomous parathyroid hyperplasia (tertiary hyperparathyroidism) to the development of a parathyroid adenoma [17]. Nonetheless, a retrospective review of 2230 patients with a history of parathyroid adenomas who received parathyroidectomy revealed that patients with vitamin D deficiency (<20 ng/mL) had a higher frequency of single-gland adenoma and not four-gland hyperplasia, as compared to those with sufficient vitamin D levels [22], arguing against the development of adenomas from hyperplasia. Therefore, the concept and pathogenesis of quaternary hyperparathyroidism remain to be further elucidated.

Reduced expression of vitamin D receptors has also been observed in parathyroid tumors [23] and in CD [24]. Decreased vitamin D signaling may contribute to the activation of cellular pathways involved in tumorigenesis, including upregulation of cyclin D1 expression [23,25]. Indeed, when transgenic mice with cyclin D1-induced PHPT were exposed to varying levels of dietary vitamin D, those receiving a vitamin D-deficient diet demonstrated accelerated parathyroid gland growth only after the onset of biochemical evidence of PHPT. These findings suggest that vitamin D deficiency promotes the enlargement of preexisting parathyroid tumors rather than initiating tumorigenesis [26]. They also suggest that parathyroid adenomas may likely be initiated independently of vitamin D deficiency. However, reduced vitamin D receptor expression in both PHPT and CD raises the possibility that vitamin D receptor dysfunction may contribute to the coexistence of these two conditions.

Many patients described in the reported cases had persistent hyperparathyroidism despite correction of vitamin D deficiency and later developed hypercalcemia following treatment with GFD. Because secondary hyperparathyroidism resulting from vitamin D and calcium malabsorption can prevent the elevation of serum calcium in patients with coexisting PHPT, the diagnosis of PHPT may be missed or delayed. Only after initiation of GFD and subsequent healing of intestinal mucosa does improved calcium absorption lead to the development of hypercalcemia, prompting further evaluation for underlying PHPT. Lastly, the development of parathyroid adenomas may be related to an autoimmune process. Autoimmune involvement of the parathyroid glands is most commonly associated with hypoparathyroidism; however, parathyroid adenomas causing PHPT have also been reported in association with other autoimmune conditions, such as rheumatoid arthritis [27,28]. Since PHPT is not considered an autoimmune disease, the lack of understanding regarding how autoimmune mechanisms may contribute to parathyroid tumorigenesis makes this association controversial.

**Figure 2:**
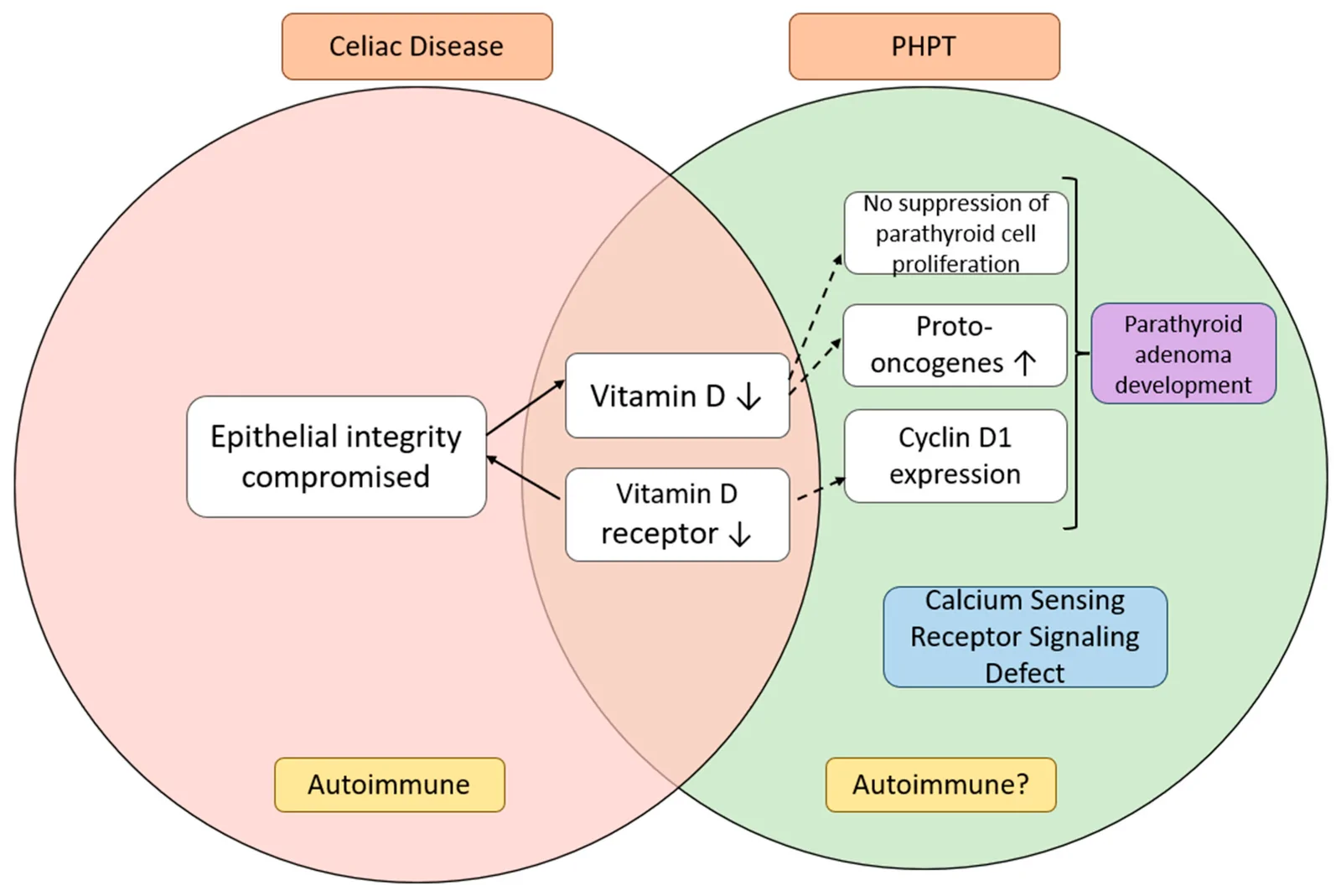
Schematic representation of the potential mechanisms linking Celiac disease (CD) and primary hyperparathyroidism (PHPT) as proposed in the literature. The dotted arrows represent a hypothetical relationship between vitamin D deficiency and possible development of parathyroid adenoma in CD. The vitamin D receptor is downregulated in both conditions and may play a role in the pathogenesis of each disease. In CD, reduced vitamin D receptor expression impairs epithelial integrity, contributing to vitamin D malabsorption. In PHPT, reduced vitamin D receptor expression upregulates cyclin D1 expression and promotes tumorigenesis. Vitamin D deficiency fails to suppress parathyroid cell proliferation and enhances proto-oncogene expression, both of which contribute to the development of parathyroid adenomas in PHPT. However, these studies limit the interpretation as they were not done in CD models. Independent coexistence of the two diseases remains a possibility. Other factors that may contribute to the pathogenesis of these diseases include autoimmunity and defects in calcium-sensing receptor signaling, particularly in PHPT.

All these observations are valuable but cannot establish that CD contributes directly to the development of parathyroid adenomas. Thus, there are no established mechanisms that explain the coexistence of PHPT and CD. However, there are proposed mechanisms in the literature, which we summarize in Figure 2. These mechanisms illustrate potential pathways through which vitamin D deficiency caused by CD may contribute to the development or progression of parathyroid adenomas, as well as the possibility that CD and PHPT may coexist independently. The current gaps in understanding the pathophysiologic relationship between CD and PHPT highlight the need for future studies to elucidate the underlying mechanisms linking these two conditions.

## 5. Normocalcemic PHPT

To add to the complexity of PHPT, a variant known as normocalcemic PHPT exists where there is a persistent elevation of PTH levels despite normal serum calcium concentration. It is a diagnosis of exclusion that should be established only after ruling out secondary causes of PTH elevation, such as vitamin D deficiency, impaired renal function, inadequate calcium intake, malabsorption, effects of certain medications, hypercalciuria, etc. The Fifth International Workshop on the evaluation and management of PHPT proposed the following diagnostic criteria for normocalcemic PHPT: normal albumin-adjusted total calcium and ionized calcium levels, and elevated intact PTH on at least two occasions over a period of 3–6 months after alternate causes of secondary hyperparathyroidism have been excluded [29,30]. Despite normal serum calcium, these patients often present with complications such as osteoporosis or nephrolithiasis. The prevalence of normocalcemic PHPT is unknown but is estimated to account for approximately 10–20% of patients with PHPT [31]. However, diagnosing normocalcemic PHPT in patients with confirmed CD can be challenging due to confounding factors, including vitamin D deficiency and resultant secondary hyperparathyroidism. Patients with persistent PTH elevation despite adequate treatment of CD, correction of vitamin D deficiency, and in the presence of normal serum calcium and phosphorus, normal renal function, and absence of medications that affect calcium regulation, may require further evaluation for normocalcemic PHPT.

Assessment of 24 h urine calcium excretion can provide additional information for biochemical evaluation. Urinary calcium is influenced by several factors, including dietary intake, intestinal absorption, bone turnover, and renal tubular handling. A normal 24 h urine calcium excretion is defined as <250 mg/day in women and <300 mg/day in men, or the weight-based cutoff is <4 mg/kg/day in both sexes. A reduced 24 h urine calcium may reflect ongoing malabsorption or inadequate calcium intake. Certain medications also affect urinary calcium excretion, including loop diuretics, thiazide diuretics, bisphosphonates, and antacids. Elevated 24 h urine calcium excretion in the presence of normal urine sodium and normal serum calcium level may support the diagnosis of PHPT; however, hypercalciuria itself can represent a cause of secondary PTH elevation, as urinary calcium is influenced by factors such as calcium intake, vitamin D status, renal function, dietary sodium intake, malabsorption, etc. [32]. In CD specifically, reduced intestinal calcium absorption may result in low urinary calcium.

**Figure 3:**
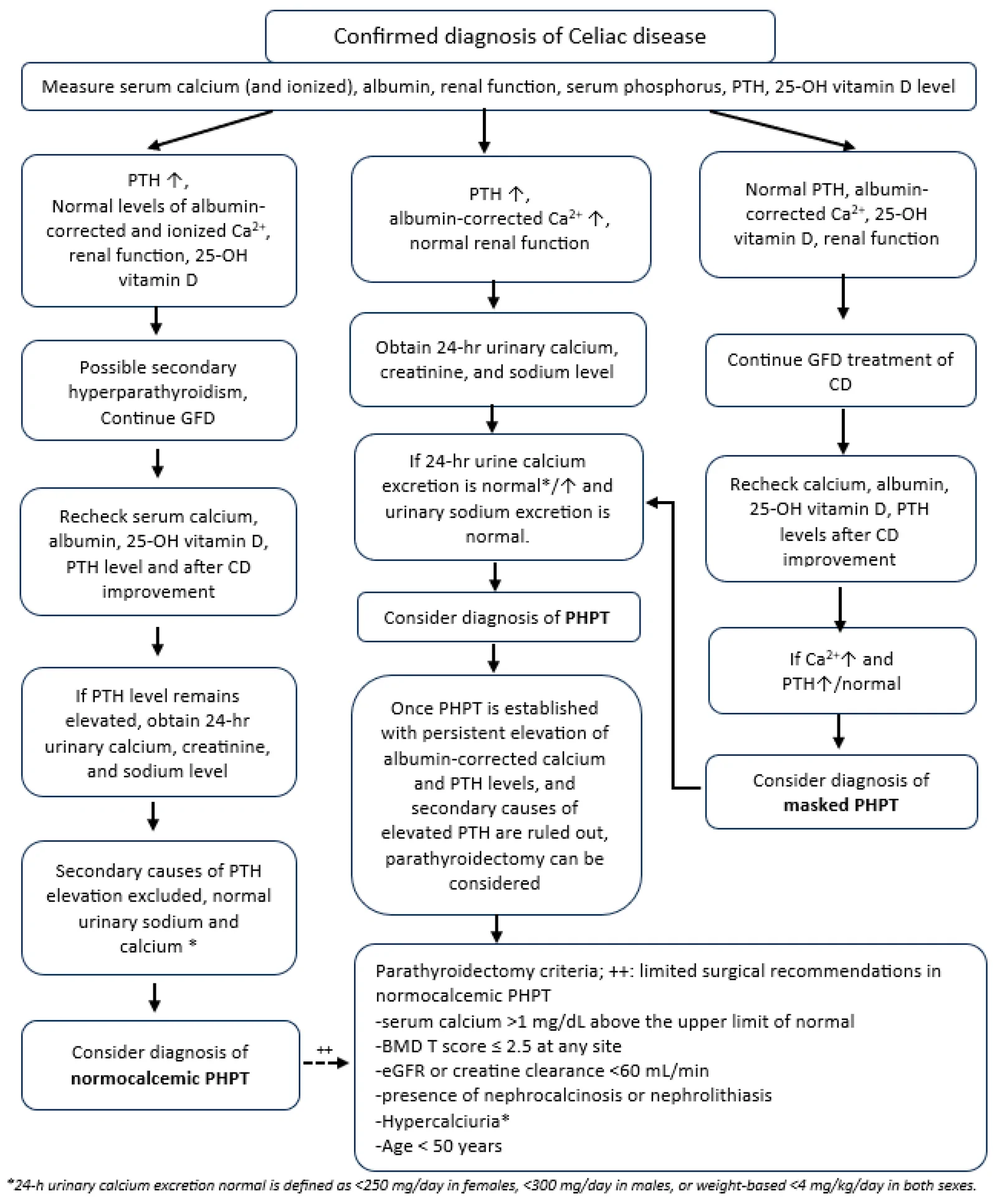
Diagnostic algorithm of hyperparathyroidism in patients with confirmed Celiac disease (CD). Normocalcemic PHPT is a diagnosis of exclusion that should be established only after ruling out secondary causes of PTH elevation, such as vitamin D deficiency, impaired renal function, inadequate calcium intake, malabsorption, effects of certain medications, hypercalciuria, etc. Ca^2+^: calcium, PTH: parathyroid hormone, 25-OH vitamin D: 25-hydroxyvitamin D, GFD: gluten-free diet, PHPT: primary hyperparathyroidism, BMD: bone mineral density, eGFR: estimated glomerular filtration rate.

The case reports and series summarized in this review, however, all demonstrated apparent elevations in serum calcium levels. Given the estimated prevalence of normocalcemic PHPT, underdiagnosis of this condition may reflect a lack of clinical awareness. Identifying patients with CD who have underlying normocalcemic PHPT is important, as untreated normocalcemic PHPT can also contribute to complications such as nephrolithiasis and osteoporosis. As with classic PHPT, surgical removal of the hyperfunctioning parathyroid gland can be considered. However, due to limited studies on the surgical management of normocalcemic PHPT, the current guidelines do not provide well-defined surgical criteria for parathyroidectomy. Nevertheless, several smaller studies have demonstrated modest improvements in quality of life and bone health following parathyroidectomy in patients with normocalcemic PHPT [33,34]. A diagnostic algorithm for hyperparathyroidism in CD patients is proposed in Figure 3.

## 6. Clinical Implications

The destruction of intestinal villi leads to malabsorption, resulting in classic symptoms such as diarrhea, bloating, abdominal pain, and weight loss. Nutritional deficiencies contribute to extraintestinal symptoms, including iron deficiency and microcytic anemia, neurological symptoms from B12 deficiency, and osteoporosis resulting from impaired calcium and vitamin D absorption [35]. Osteopenia and osteoporosis have been reported in up to 70% of patients with CD at the time of diagnosis [36]. Despite its high prevalence, the role of dual-energy X-ray absorptiometry (DEXA) scanning for assessment of bone health in patients with CD at diagnosis or during follow-up is not clearly defined in the most recent American College of Gastroenterology Guideline Update [37].

In patients with CD, secondary hyperparathyroidism is considered a major contributor to BMD loss. Impaired nutrient absorption in the small intestine results in decreased absorption of calcium and vitamin D, potentially leading to hypocalcemia and vitamin D insufficiency or deficiency. This subsequently stimulates PTH secretion, resulting in secondary hyperparathyroidism. Excess PTH promotes accelerated bone resorption and may contribute to bone loss [38]. Serum PTH levels correlate with markers of bone formation (e.g., osteocalcin) and bone resorption (e.g., telopeptides of type I collagen, ICTP), indicating increased bone turnover [39]. A cross-sectional study of 387 adults with CD found that 27% had elevated PTH levels, and increased PTH concentrations were significantly associated with reduced BMD [40]. Although secondary hyperparathyroidism is a well-established mechanism contributing to CD-associated bone loss, the precise relationships among the degree of malabsorption, vitamin D status, PTH levels, mucosal recovery, timing of GFD initiation, and recovery of bone density remain incompletely understood [39].

## 7. Gluten-Free Diet and Celiac Disease

A GFD remains the cornerstone and most effective therapy for patients with CD. It is currently the only intervention consistently shown to promote mucosal healing, restore nutrient absorption, and prevent long-term complications such as MBD and secondary hyperparathyroidism [41,42,43,44]. Initiation of a strict GFD can reverse the metabolic consequences of malabsorption. By promoting recovery of the intestinal mucosa, a GFD improves calcium and vitamin D absorption, normalizes serum levels of these nutrients, and subsequently reduces PTH secretion. Nonetheless, if PTH levels remain elevated despite improvement in intestinal mucosal integrity and nutrient absorption, this may suggest the presence of previously undiagnosed PHPT. In other words, GFD does not cause PHPT; rather, it may unmask a pre-existing, coexisting PHPT.

Clinical studies have demonstrated that measurable improvements in BMD can occur as early as the first year of adherence to GFD, highlighting the relatively rapid improvement in bone metabolism following correction of malabsorption [44,45,46,47]. However, the extent of BMD recovery is influenced by several factors that can affect bone health, including age, menopausal status, duration of untreated CD prior to diagnosis, adherence to GFD, nutritional status, etc. Importantly, the prevalence of hypovitaminosis D and secondary hyperparathyroidism is significantly lower among patients who achieve histological response to GFD compared to those who are newly diagnosed or fail to achieve mucosal recovery [46]. These findings further support the fact that strict adherence to gluten avoidance has beneficial effects not only on intestinal healing but also on systemic bone health.

Despite these improvements, important limitations remain. A subset of patients continues to demonstrate residual bone deficits even after years of adherence to a GFD. These deficits are mostly observed at peripheral skeletal sites and may reflect incomplete recovery or irreversible bone loss that occurred prior to CD diagnosis [47]. In particular, the distal one-third radius is a commonly assessed peripheral skeletal site in patients with hyperparathyroidism [48]. Persistent deficits in BMD recovery among some patients may suggest additional contributing factors that affect bone health, including the possibility of coexisting primary hyperparathyroidism.

## 8. Conclusions

Both hyperparathyroidism and CD adversely affect bone health and are important contributors to osteoporosis. Although elevated PTH levels in patients with CD are typically attributed to secondary hyperparathyroidism, multiple case reports and case series suggest a possible association between CD and PHPT. The relationship between these two conditions remains poorly understood. A subset of patients with CD continues to experience impaired bone health despite treatment with GFD, suggesting the presence of additional contributing factors that affect bone health, including PHPT. Given the lack of definitive recommendations regarding bone health screening or follow-up with DEXA scan in patients with CD, accurate diagnosis is essential to guide appropriate management and surveillance. CD is managed with GFD, whereas the definitive treatment for PHPT is surgical intervention with parathyroidectomy.

Furthermore, PHPT can result in several clinically significant complications beyond its effects on bone health, including renal, gastrointestinal, cardiovascular, and neuropsychiatric manifestations. Therefore, it is important to recognize that PHPT may coexist with CD as a distinct pathological process, which may initially be masked by calcium and/or vitamin D malabsorption and the resulting secondary hyperparathyroidism. Population-based studies have demonstrated an association between CD and PHPT, especially within the first five years following CD diagnosis, although causality remains unproven. Persistent or recurrent elevation in PTH despite correction of vitamin D deficiency should prompt reassessment for PHPT after excluding other secondary causes of PTH elevation. This approach may help identify previously unrecognized or masked PHPT, facilitate timely diagnosis, and potentially prevent further loss of BMD. Lastly, this review summarizes recent observational data to raise awareness that persistent hyperparathyroidism in CD should not automatically be attributed to malabsorption alone.

## Figures and Tables

**Table 1:** Baseline characteristics of patients with Celiac disease as reported in case series and reports. This represents a wide spectrum of age groups with evidence of bone abnormalities and parathyroid adenomas (2002–2024).

Case Reports	Patient Characteristics Age (Years)/Sex	Osteoporosis	Calcium (mg/dL)	PTH (pg/mL)	25-OH Vitamin D (>30 ng/mL)	Location of Parathyroid Adenoma
**Freeman** **et al. 2024 [9]**	41/F	Present	10.5 * (8.02–10.42)	68 * (12.3–50.9)	not reported	Right inferior
**Stieben et al. 2023 [10]**	49/F	Present	12.2 ^†^ (8.5–10.5)	131 ^†^ (15–65)	36 ^†^	Right inferior
**Fayadh et al. 2020 [11]**	40/F	Present	14.2 (8.6–10)	241.4 (15–65)	35.7	Left inferior
**Anaforoglu et al. 2012 [12]**	14/F	Unknown	13.4 ^†^ (8.2–10.6)	955 ^†^ (11.1–79.5)	7 ^†^	Right inferior
**Wu et al. 2012 [13]**	45/F	Present	10.8 * (8.4–10.2)	393 (11–64)	31	Left inferior
	42/F	Present	10.4 * (8.4–10.3)	134 (11–64)	not reported	Single, not specified
**Fanciulli et al. 2011 [14]**	35/F	Unknown	11.1 (8.4–10.8)	290 (10–60)	26	Left superior
**Alzahrani et al. 2008 [15]**	24/F	Present	10.8 (8.4–10.4)	325 (15–65)	58	Right inferior
**Maida et al. 2006 [4]**	72/F	Present	11.2 (8.8–10.4)	55 (8.5–50.9)	26	Left inferior
	48/F	Present	10.6 (8.8–10.4)	125 (8.5–50.9)	27.6	Left superior
	47/F	Unknown	10.8 (8.8–10.4)	54 (8.5–50.9)	33	Right inferior
	80/F	Unknown	11.4 (8.8–10.4)	176 (8.5–50.9)	not reported	Left inferior
	70/F	Unknown	11.3 (8.8–10.4)	68 (8.5–50.9)	20.9	Right superior
	87/F	Unknown	10.6 (8.8–10.4)	110 (8.5–50.9)	16.7	Right superior
	59/F	Unknown	10.8 (8.8–10.4)	138 (8.5–50.9)	17.9	Left superior
**Singh et al. 2002 [16]**	32/F	Unknown	11.8 (8.9–10.5)	354 (10–65)	normal	Right inferior
**Brain et al. 2004 [17]**	32/F	Present	12.5 (8.6–10.2)	1414.5 (10.4–65.1)	7	Right inferior

*Note*: “Normal” reflects the normal value as stated in the article. “Unknown” is used when the condition was not mentioned at all in the original article. “Not reported” is used when the serum level was mentioned but the actual value was not reported. All calcium, PTH, and 25-OH vitamin D levels were reported on reassessment after post-GFD initiation unless otherwise stated. ^†^ Values were given prior to GFD as the initial diagnosis was PHPT, which subsequently led to a diagnosis of CD. * Values were originally reported as mmol/L for calcium and pmol/L for PTH levels.

## Data Availability

No new data were created or analyzed in this study. Data sharing is not applicable to this article.

## Abbreviations

The following abbreviations are used in this manuscript:

CD	Celiac Disease
MBD	Metabolic Bone Disease
BMD	Bone Mineral Density
GFD	Gluten-Free Diet
PHPT	Primary Hyperparathyroidism
PTH	Parathyroid Hormone
CaSR	Calcium-Sensing Receptor
DEXA	Dual-Energy X-ray Absorptiometry
ICTP	Cross-Linked C-Terminal Telopeptide of Collagen Alpha-1 (I) Chain
